# Comparisons of fall armyworm haplotypes between the Galápagos Islands and mainland Ecuador indicate limited migration to and between islands

**DOI:** 10.1038/s41598-021-83111-5

**Published:** 2021-02-10

**Authors:** Rodney N. Nagoshi, Joanna Lizeth Allauca Vizuete, M. Gabriela Murúa, Sandra Garcés-Carrera

**Affiliations:** 1 Center for Medical, Agricultural and Veterinary Entomology, United States Department of Agriculture-Agricultural Research Service, Gainesville, FL USA; 2 Ministerio de Agricultura y Ganadería (MAG), Dirección Distrital (05D01), Cotopaxi, Ecuador; 3 Estación Experimental Agroindustrial Obispo Colombres (EEAOC), Consejo Nacional de Investigaciones Científicas y Técnicas (CONICET), Instituto de Tecnología Agroindustrial del Noroeste (ITANOA), Las Talitas (T4104AUD), Tucumán, Argentina; 4 National Institute of Agriculture Research (INIAP), Quito, Ecuador

**Keywords:** Animal migration, Genetic markers, Genetic variation

## Abstract

The migration of the fall armyworm (*Spodoptera frugiperda*) is of topical interest because of its recent introduction and rapid dissemination throughout the Eastern Hemisphere. This study compares fall armyworm from island and mainland locations in Ecuador to estimate migration behavior. The Galápagos Islands is a province of Ecuador whose mainland coast lies approximately 1000 km to the west and is the closest major land mass. Air transport modeling indicates that natural migration from the mainland to the Galápagos is unlikely, suggesting that most, if not all, the introgressions of mainland fall armyworm into the Galápagos are occurring through trade-assisted transport in contaminated cargo, which is offloaded at the Galápagos port of entry in San Cristóbal island. Haplotype studies are consistent with this limited migration and further show divergence in the fall armyworm from San Cristóbal with those from the neighboring island of Santa Cruz despite their close proximity (less than 100 km distance) and favorable winds for inter-island flights. These observations indicate that water poses a significant barrier for moth migration in this region, with human-assisted transport probably playing a more important role than natural migration.

## Introduction

A useful model organism for studying the migration of lepidopteran pests is the noctuid moth *Spodoptera frugiperda* (J. E. Smith), commonly known as the fall armyworm. Native to the Western Hemisphere, fall armyworm is a major pest of corn (*Zea mays* L.) and frequently causes economic damage to sorghum (*Sorghum vulgare* Pers*.*), cotton (*Gossypium hirsutum* L.), and several turf grass species^1^. For more than a decade, effective control of this pest in the Western hemisphere has largely depended on genetically modified maize hybrids expressing *Bacillus thuringiensis* (Bt) insecticidal proteins. However, significant resistance to some Bt products was observed in Puerto Rico and subsequently found in other locations in North and South America^2–6^. This has led to increased interest in understanding fall armyworm genetic diversity and gene flow among its populations and the possible exchange of resistance alleles between geographically distant populations.

The migratory capabilities of fall armyworm is of interest because its discovery in western Africa in 2016 was rapidly followed by infestations throughout that continent, then into India, Southeastern Asia^7–10^, and most recently Australia^11^. Although the pest is now widely distributed in the hemisphere, the long-term consequences to food security will depend on how permanent fall armyworm populations end up being distributed and their migratory behavior from these sites. With respect to the latter, it will be important to know the relative contributions of natural migration versus that which occurs through human-assisted transport (e.g., contaminated cargo), particularly with respect to traversing natural physical barriers such as deserts, mountains, and bodies of water.

Long-distance migration of fall armyworm is best characterized in North America. The pest is intolerant to prolonged freezing conditions, which limits the distribution of known winter populations to Mexico, the Caribbean, and the southern portions of Florida and Texas^12–14^. Annual northward migrations begin in the spring with movements of large populations to southern Canada, a journey of several thousands of kilometers occurring over successive generations. Fall armyworm has only been observed to fly nocturnally, with sustained flight durations of no more than 12 h^1,15,16^. If this truly represents the upper limit of sustained flight, then landing sites are required during the daytime rest period, limiting migration over water to that which can be traversed in a single night flight. The timing and direction of the migratory flights in North America are dictated by regional air transport systems that strongly favor northward flight during the spring and summer months^15,17^. Analogous long-distance migration in other locations, such as the Caribbean or South America, is plausible but remains uncertain.

A productive method for inferring fall armyworm migration behaviors involves genetic comparisons using a well-characterized set of mitochondrial and nuclear markers that can subdivide populations by geography and host plant usage. Genetic studies identified two populations of fall armyworm that are morphologically indistinguishable at all stages but differed in their host distribution^18,19^. These are designated as the C-strain, which is preferentially found in corn and sorghum, and the R-strain, which predominates in pastures, turf grasses, and alfalfa^18–22^. Genetic markers that can distinguish the two strains include the mitochondrial *Cytochrome Oxidase Subunit I* (*COI*) and the sex-linked *Triosephosphate isomerase* (*Tpi*) genes^23,24^. A segment of *COI* carries polymorphisms that in addition to differentiating the two strains also identifies two C-strain groups with different geographical distributions in the Western Hemisphere^14,25,26^. Equivalent markers have not yet been found for the R-strain. Strain-specific polymorphisms within the *Tpi* gene have been used to confirm *COI* strain identification^24,27^, identify hybridization between strains^8,24,28^, and to compare populations from different locations^29,30^.

The two strains are considered to be sympatric, differing in relative frequency depending on host and habitat but with populations at defined locations in North America, Brazil, and Argentina rarely exclusive to a single strain^21,22,31,32^. This suggests some plasticity in host usage, consistent with laboratory studies showing both strains are capable of developing on many different hosts^20,33–35^. If this is true in the field, then the observed biased distribution of the two strains reflects strain-specific preferences rather than an absolute requirement for different host types. However, there appear to be some notable exceptions to sympatry. It was recently found that fall armyworm collected from both C-strain and R-strain preferred host plants at multiple sites in Ecuador were > 99% C-strain by both *Tpi* and *COI* markers, indicating the near absence of the R-strain in this country and therefore regional differences in the migration of the two strains. In addition, a complicated picture is emerging in the Eastern Hemisphere, where discrepancies between genetic markers suggest populations dominated by the C-strain and interstrain hybrids, with the R-strain virtually absent^8^.

The *COI* and *Tpi* suite of markers was recently used to characterize populations in the northern portion of South America, which showed strong similarities with fall armyworm from Brazil and Argentina and substantial differences with those from the Caribbean and the United States^21,36,37^. The results suggested limited interactions between populations in the two Americas and, specifically, that the movement of fall armyworm from the mainland to Caribbean islands and between islands occurred infrequently enough to identify genetic differences between locations^38^. In this paper, we extend these studies to include the Galápagos Islands, which lies in the Pacific Ocean about 1000 km from mainland Ecuador, the nearest major land mass. Comparisons between the Galápagos and mainland fall armyworm using the *COI* and *Tpi* markers were combined with modeling of high-altitude dispersion based on regional wind patterns to address the probable origin of the Galápagos fall armyworm and to make inferences about how much natural migration might be occurring between the sampling sites. The results indicate that human-assisted migration of fall armyworm through trade is likely to be the major contributor of mainland fall armyworm entering the Galápagos, as well as for population movements between islands, which could have policy implications concerning the degree and frequency of cargo inspections. In addition, the data add to past studies indicating that bodies of water provide a significant migration hurdle even between relatively close land masses.

## Results

### Host strain analysis of the Galápagos population

**Figure 1:**
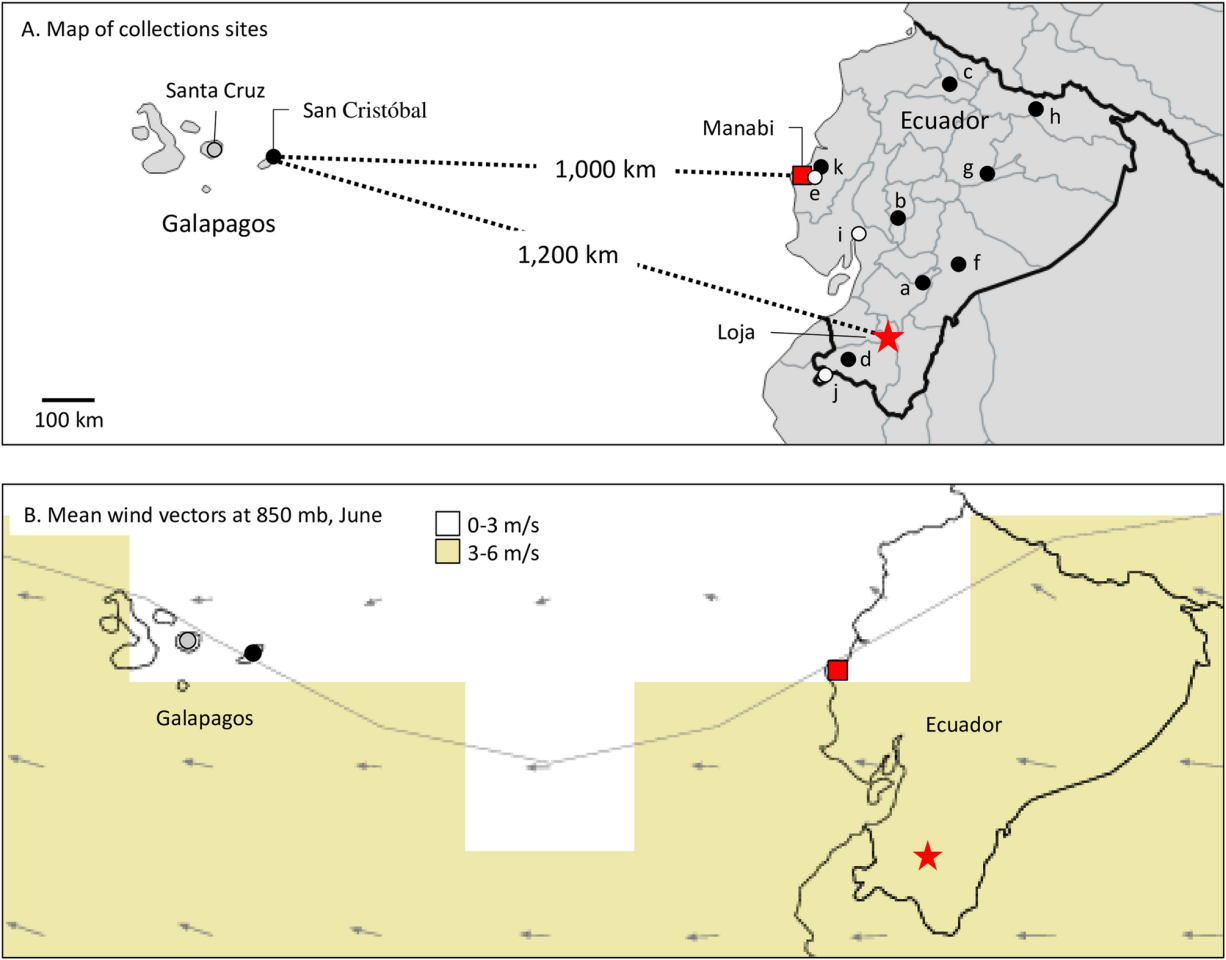
Maps showing locations of fall armyworm sampling sites in the Galápagos islands and mainland Ecuador as described in Table 1. (**A**) Map of Ecuador displaying outline of provinces and approximate locations of sampling sites from corn (black circles) and rice (white circles) host plants. In red are locations used as origins for HYSPLIT analysis. The map was created using Quantum Geographic Information System (version 2.18.2, http://qgis.osgeo.org). (**B**) Average wind vectors for the month of June at 850 mb (see Wind Climatology Maps in “Methods”). The map was obtained online from the International Research Institute for Climate and Society (Earth Institute, Columbia University, New York, NY; http://iridl.ldeo.columbia.edu/maproom/Global/Climatologies/Vector_Winds.html).

**Figure 2:**
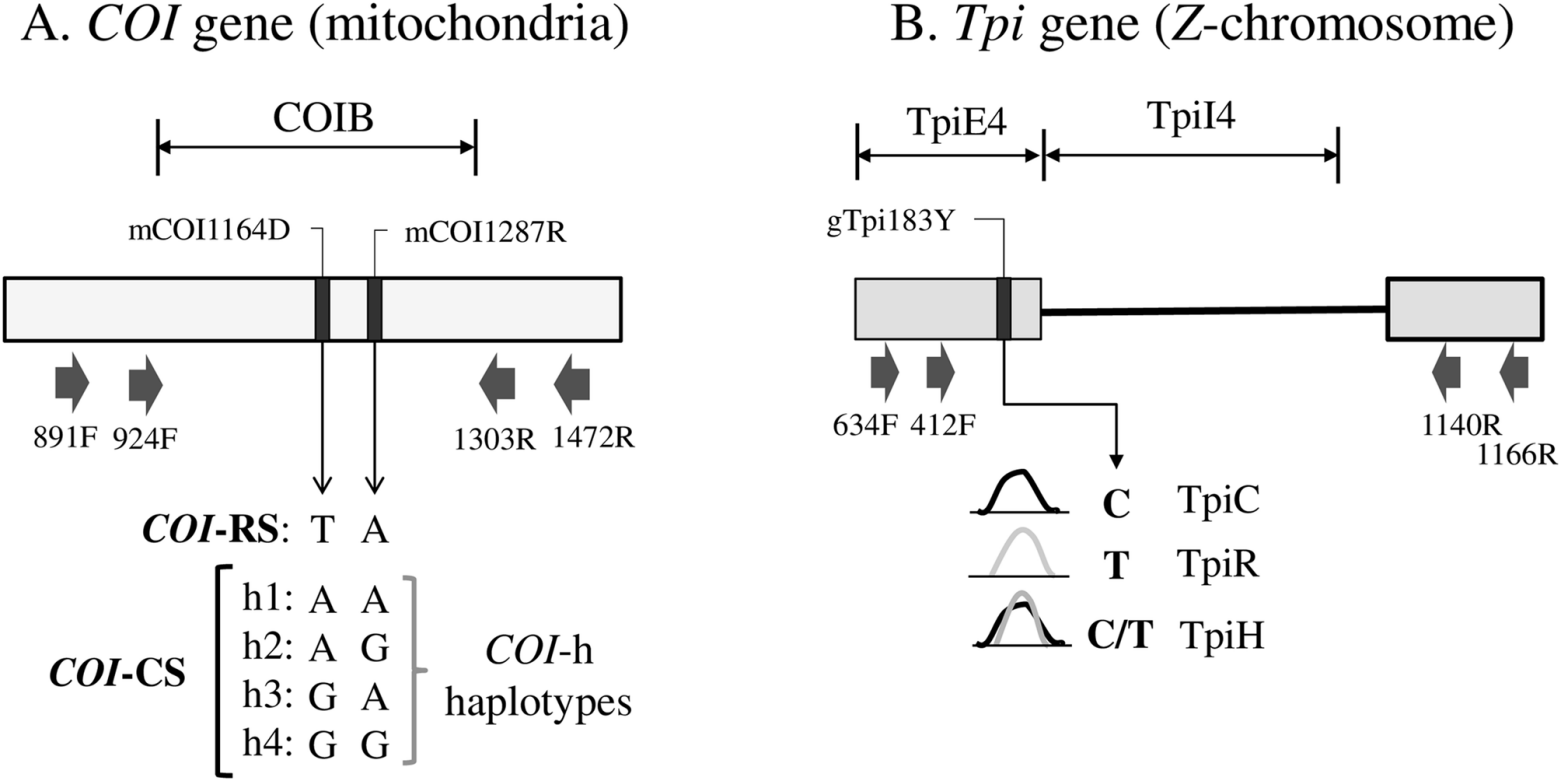
Diagrams of the segments from the *COI* and *Tpi* genes used for the genetic analysis (derived from^42^). (**A**) COIB gene segment identifying PCR primers used to amplify the fragment (block arrows) and the strain and *COI*-h haplotype defining the mCOI1164D and mCOI1287R polymorphic sites. *CO1*-RS is defined as a T and A at mCOI1164D and mCOI1287R, respectively. There are four corn-strain (*CO1*-CS) haplotypes (h1–h4) with an A or G observed at both mCOI1164D and mCOI1287R. (**B**) Portion of the fall armyworm *Tpi* gene with block arrows indicating PCR primers. The TpiE4 exon segment contains the gTpi183 site that is polymorphic for a C or T. Representative DNA chromatograph patterns are shown to illustrate how TpiC, TpiR, and TpiH are defined. The TpiI4 intron segment lies adjacent.

**Figure 3:**
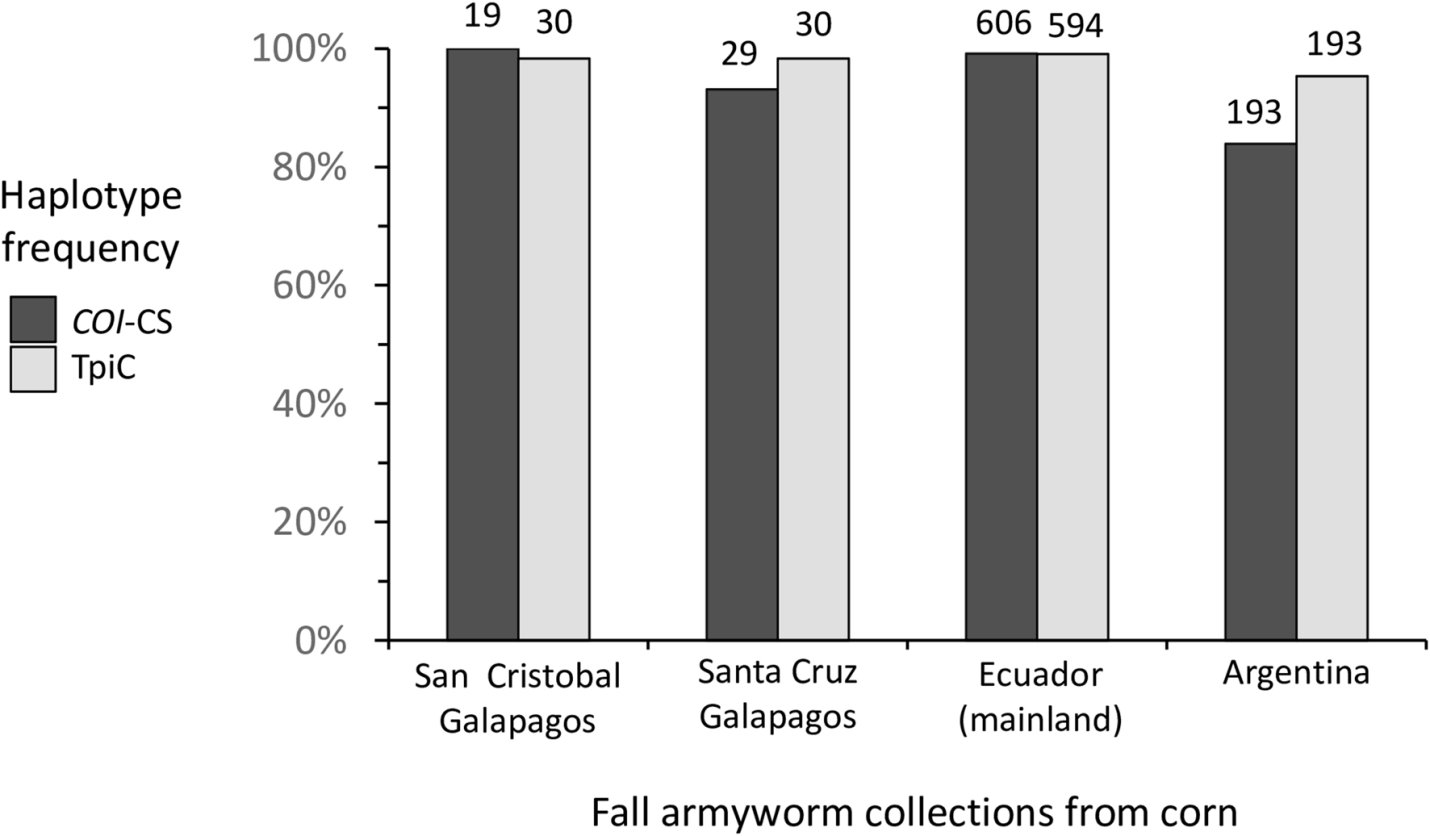
Frequency distributions of the C-strain *COI*-CS and TpiC haplotypes. Specimens are fall armyworm larvae collected from corn from two sites in the Galápagos. These were compared to specimens previously collected from Argentina corn^21^, and from corn and rice in mainland Ecuador^42^. Numbers above columns indicate number of specimens analyzed.

Fall armyworm larvae were collected from corn hosts from two islands in the Ecuador province of the Galápagos, Santa Cruz and San Cristóbal, and compared with those from multiple locations in Ecuador (Fig. 1A). These were initially analyzed for strain identity using polymorphisms in a segment of the mitochondrial *COI* gene, COIB (Fig. 2A). The great majority of specimens (44/46) expressed the C-strain haplotype, *COI*-CS, with only two *COI*-RS found, both on the island of Santa Cruz (Fig. 3). This predominantly C-strain identification was confirmed using markers from the nuclear *Tpi* gene segment, TpiE4 (Fig. 2B), where 98% of Galápagos specimens expressed the C-strain TpiC marker (Fig. 3). This predominantly C-strain composition on both islands is similar to that observed for fall armyworm collected from corn hosts in mainland Ecuador and Argentina.

### *COI*-h haplotype composition

**Figure 4:**
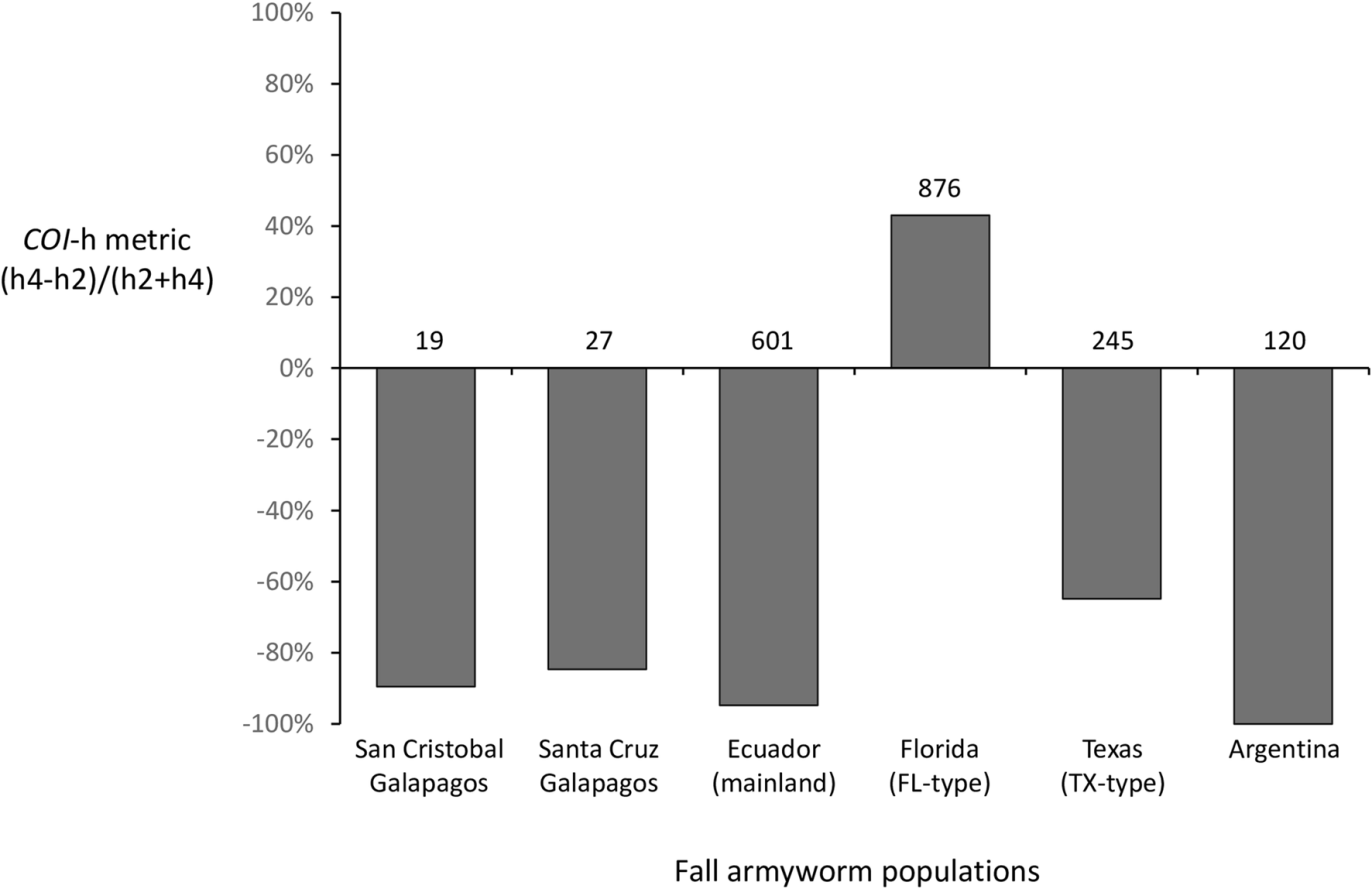
Frequency distributions of the COIB h-haplotype h2 relative to h4 for Ecuador and select locations in the Western Hemisphere. The relative frequency of h4 is calculated by the equation (h4 − h2)/(h2 + h4) presented as a percentage. Numbers indicate the number of *COI*-CS specimens analyzed. Collections from mainland Ecuador, Florida, Texas, and Argentina were previously described^29,42^.

The COIB segment contains *COI*-h polymorphisms that subdivide the C-strain into four haplotype categories, two of which (h2 and h4) display regional differences in relative frequency (Fig. 2A)^36^. The *COI*-h metric for the Galápagos sites were similar to that observed for fall armyworm populations from the Ecuador mainland as well as Argentina, all with strong negative values indicative of the TX-type *COI*-h haplotype profile (Fig. 4). In contrast, the FL-type population found in the Caribbean and southeastern United States exhibited a strongly positive metric.

### TpiI4 haplotype comparisons

**Figure 5:**
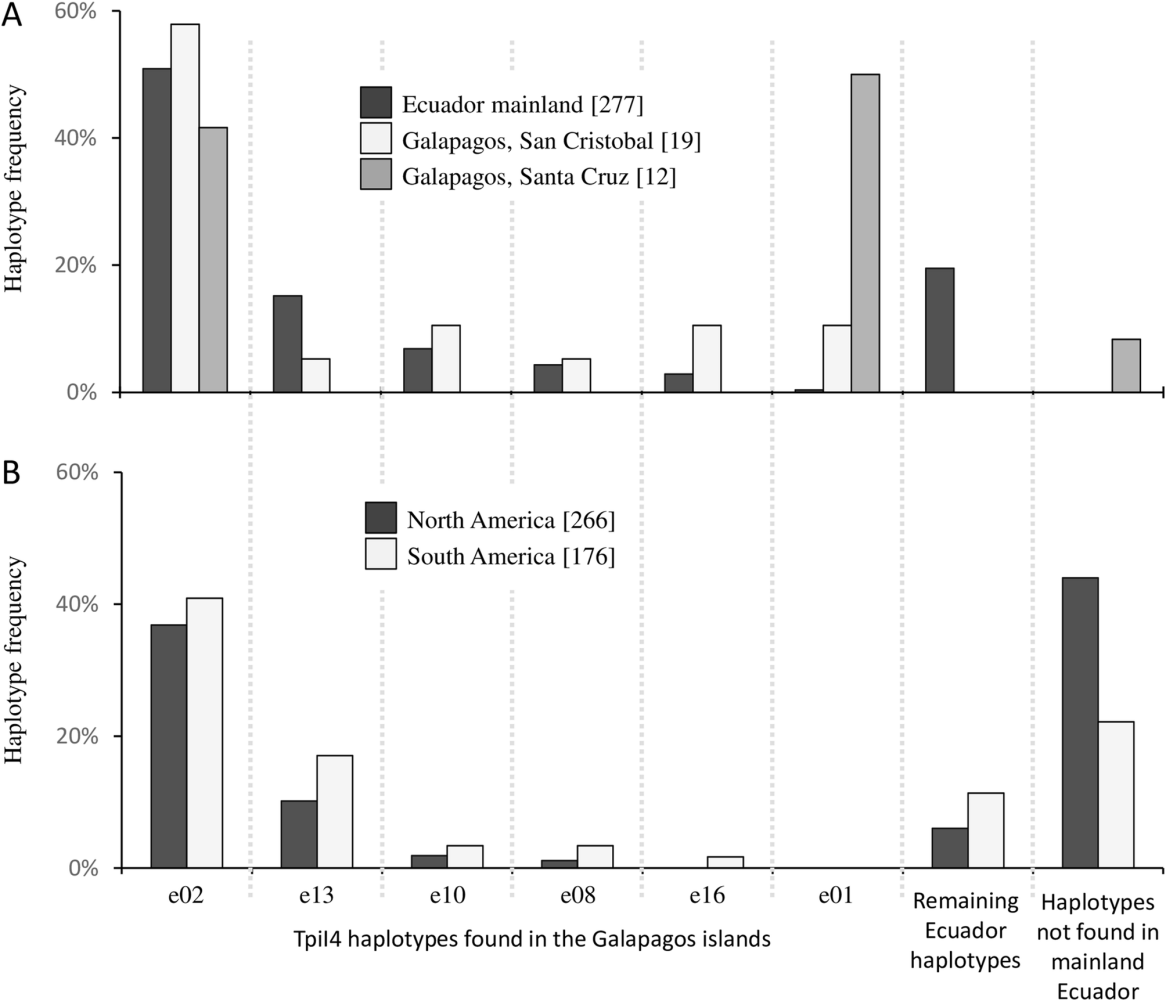
Bar graph showing frequencies of the TpiI4 haplotypes found in Ecuador in different collections. The number of sequences examined is in brackets. The X-axis lists haplotype categories found in mainland Ecuador including four also found in the Galápagos. The mainland Ecuador haplotypes not found in the Galápagos are pooled. The final category is made up of haplotypes not found in Ecuador but detected elsewhere. (**A**) Comparisons of haplotype data from mainland Ecuador and the Galápagos. (**B**) Comparisons of mean haplotype frequencies from Florida and Texas (North America) and Peru, Bolivia, Argentina, and Brazil (South America).

The *Tpi* intron (TpiI4) segment immediately following the TpiE4 exon is highly polymorphic and thereby provides a potentially useful tool to compare populations (Fig. 2B)^29,30^. In the five locations compared (Fig. 5), an average of 51% of the sequences obtained were heterozygous for the TpiI4 segment as defined by the presence of one or more polymorphic sites and were not analyzed. The remaining sequences from specimens homozygous or hemizygous for TpiI4 could unambiguously be categorized by haplotype. From the Ecuador mainland a total of 19 TpiI4 sequence variants were identified of which six have been found to date in the Galápagos island of San Cristóbal (Fig. 5A). These follow a similar frequency profile as found in the mainland with haplotype e02 predominating. A different profile was observed in the neighboring Galápagos island of Santa Cruz where 50% of the specimens carried the TpiI4 haplotype e01, which was found only once in the 277 specimens analyzed from mainland Ecuador. One specimen had a sequence not found in the mainland collections with the remainder expressing the most frequent e02 haplotype. Pearson *r* analysis of the haplotype profile produced by the TpiI4 haplotypes found in the Galápagos (e01, e02, e08, e10, e13, and e16) showed a significant positive correlation between mainland Ecuador and San Cristóbal (r = 0.931, P = 0.007) but not between the mainland and Santa Cruz (*r* = 0.400, *P* = 0.432) or San Cristóbal and Santa Cruz (*r* = 0.590, *P* = 0.217).

The Galápagos TpiI4 haplotype frequencies in pooled collections from the United States and South America (excluding Ecuador) showed a similar profile with that found in populations from the Ecuador mainland with a plurality expressing e02 followed by e13, with the remaining Ecuadorean haplotypes below 5% (Fig. 5B). The Ecuador populations contain most of the TpiI4 variation found on the continent, as only 22% of the pooled collections from Argentina, Bolivia, Brazil, and Peru carried haplotypes not found in Ecuador. This contrasts with the United States collections where the frequency of this category was 44%. The TpiI4 e01 haplotype is so far limited to Ecuador, with high frequencies observed only in the Galápagos Islands.

**Figure 6:**
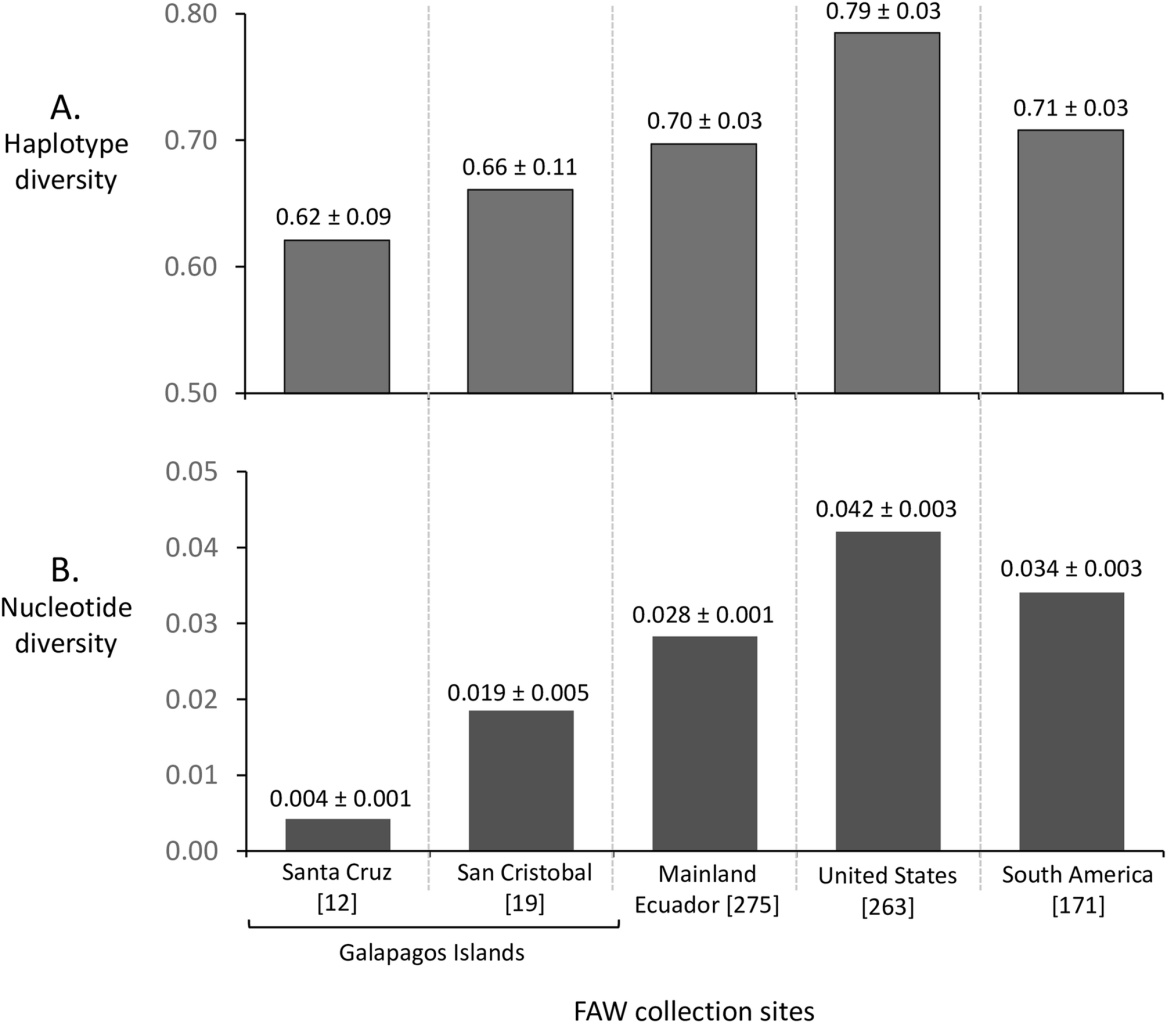
Measurements of genetic variation between select locations in Ecuador and the rest of the Western Hemisphere. (**A**) Haplotype diversity calculations (± standard deviation). (**B**) Nucleotide diversity metric (± standard deviation). Numbers in parentheses indicate sequences analyzed.

Genetic variation as measured by haplotype diversity was similar in all Ecuador locations, ranging between 0.6 and 0.7, with the highest value found in mainland populations followed by those in San Cristóbal and with the lowest value in the neighboring island of Santa Cruz (Fig. 6A). The same profile was observed using nucleotide diversity as a metric of variation but with larger differences between locations. In this case nucleotide diversity in the Santa Cruz collections was more than fourfold lower than those from San Cristóbal and sevenfold lower than mainland fall armyworm (Fig. 6B). The genetic variation in the Ecuador mainland was comparable to that calculated for pooled collections from South America and lower than that observed in the United States as measured by both metrics.

### Assessing the likelihood of natural migration to and within the Galápagos

**Figure 7:**
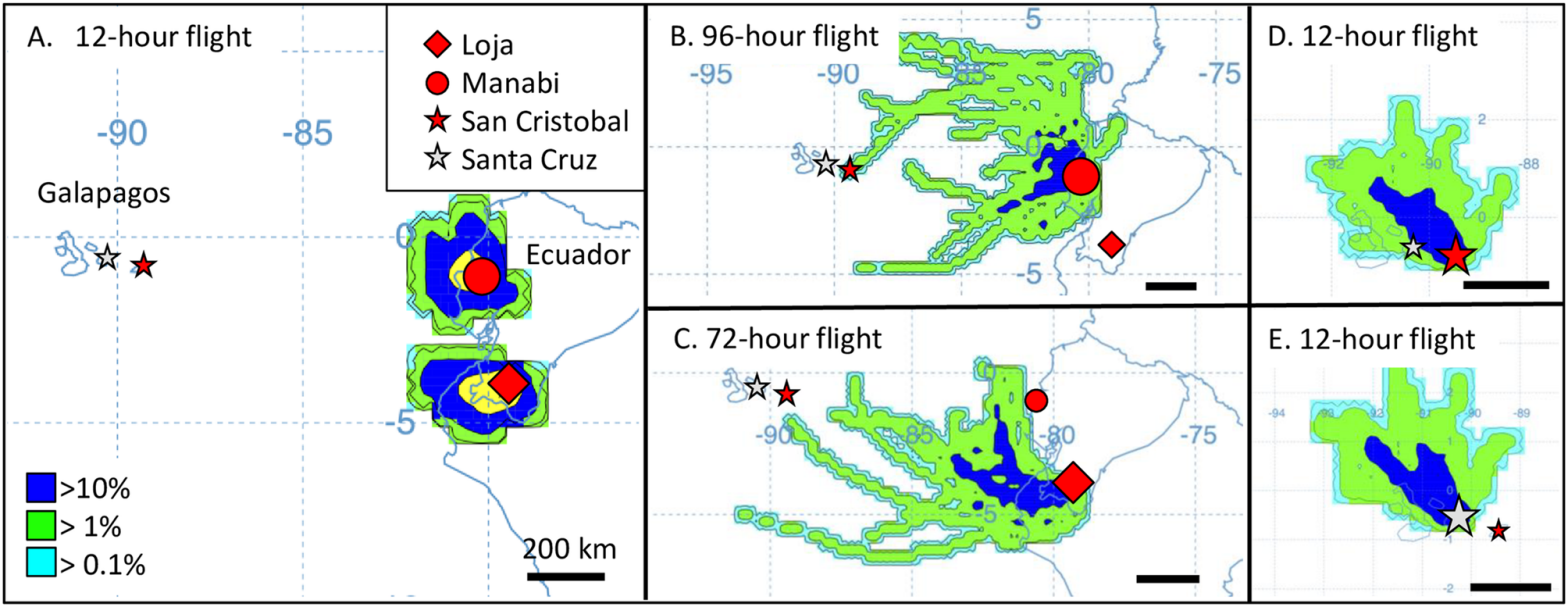
HYSPLIT forward projections of dispersions from locations in Ecuador based on 2019 air transport data for the month of June. Thirty projections of either 12-h, 72-h, or 96-h continuous flight durations were plotted. The frequency that a projected trajectory passed over a grid cell was summed and then normalized by the total number of trajectories to give the color-coded percentages. Relevant locations are identified by colored shapes, which when enlarged are origins for the HYSPLIT modeling. Maps were obtained using HYSPLIT modeling from the Air Resources Laboratory (ARL) READY web site run by NOAA (http://ready.arl.noaa.gov/HYSPLIT.php^49^. (**A**) HYSPLIT 12-h continuous flight projection originating from Loja (− 4.02, − 79.27) and Manabi (− 1.10, − 80.43) provinces in mainland Ecuador. (**B**) 96-h continuous flight projection from Manabi province. (**C**) 72-h continuous flight projection from Loja Province. (**D**) 12-h projection from San Cristóbal island (− 0.87, − 89.43). (**E**) 12-h projection from Santa Cruz island (− 0.59, − 90.32).

The Galápagos islands are approximately 1200 km from Ecuador, which is the closest major land mass and therefore the most likely source of fall armyworm arriving by natural migration. There are two partially overlapping corn seasons in Ecuador with the first harvest period from May to July and the second from August through November. We anticipate that peak fall armyworm populations would occur during these time periods. An examination of average monthly wind patterns identified June as among the most favorable for westward migration (Fig. 1B). We used HYSPLIT to project wind-directed dispersion from major cornfields in the Loja Province based on 12 h of sustained flight, which approximates the nocturnal migration behavior of fall armyworm observed in North America^17^. Flight projections were repeated once a day for the month of June 2018 and the data combined in a frequency map of the extrapolated dispersion pattern. The projections show that a 12-h flight time was insufficient to reach the Galápagos, with projections originating from either Manabi or Loja provinces ending about 850 km from the islands (Fig. 7A). Continuous flights of at least 72 h were required for projections to reach the vicinity of the Galápagos from the Ecuador mainland (Fig. 7B,C).

Natural migration is more plausible within the Galápagos islands themselves, specifically between the collection sites on San Cristóbal and Santa Cruz islands that are separated by only approximately 100 km of water. HYSPLIT projections originating from San Cristóbal show a high frequency of pathways overlapping Santa Cruz as well as other islands in the area (Fig. 7D). Projections originating from Santa Cruz suggest that June winds favor transport to the islands to the northwest, while movement to San Cristóbal to the east is not supported (Fig. 7E).

## Discussion

The COIB and *Tpi* haplotype data are consistent with the Galápagos fall armyworm being derivative of populations from mainland Ecuador. The San Cristóbal island specimens, which were isolated from corn, predominantly expressed the expected C-strain *COI*-CS and TpiC markers and were of the TX-type *COI*-h haplotype category to approximately the same degree as fall armyworm from mainland Ecuador and the rest of South America (Fig. 4). Most compelling is that the TpiI4 variants found in San Cristóbal are a subset of those found in mainland Ecuador with a similar haplotype frequency profile (Fig. 5A). We believe this suggests significant and consistent movement of fall armyworm from the mainland to the Galápagos.

However, HYSPLIT analysis of migration potential based on air transport systems indicate that the movement of fall armyworm from mainland Ecuador to the Galápagos by natural migration is unlikely. Projections based on average monthly wind patterns estimate that at least 48 h of sustained flight would be required to traverse the approximately 1200 km needed to reach the Galápagos islands. Since fall armyworm has only been observed to fly nocturnally, this would be well beyond the 8–12-h flight time normally observed for this species during the North American migration season. It is possible that more extreme weather conditions, such as severe storms, could provide strong enough wind vectors to allow fall armyworm to survive such a journey. However, such events would likely be sporadic and involve much smaller numbers than typically associated with mass migrations.

Given these observations we believe that the most important mechanism for interactions between mainland Ecuador and Galápagos fall armyworm populations is human-assisted migration through the trade of contaminated goods. There is evidence that a number of exotic insect species have been introduced into the Galápagos islands by this route^39^. It is estimated that about 75% of the agricultural products used in the Galápagos are sent from the port city of Guayaquil in Ecuador, with reports that as of 2010 pest inspections on these cargo transports were minimal^40^. Because San Cristóbal is the commercial port-of-entry for the Galápagos, fall armyworm entering by this process would be expected to first become established locally. While the size of any single introgression would be small in numbers, occurrence on a regular basis would significantly contribute to the local population. Under this scenario, the San Cristóbal fall armyworm haplotype profile should be similar to that measured on the mainland, just as we observed (Fig. 5).

The island of Santa Cruz is within 100 km of San Cristóbal, well within the single-night flight range of fall armyworm based on HYSPLIT analysis (Fig. 7D). In addition, there is substantial boat traffic between the islands providing a means for human-assisted insect transport by that route^40,41^. Because of these opportunities for inter-island movements, we expected the Santa Cruz fall armyworm to be genetically similar to those on San Cristóbal. This was not the case with the majority TpiI4 haplotype in Santa Cruz (Ecu01) not found in San Cristóbal (Fig. 5A). In fact, this haplotype was found in only a single specimen out of 220 tested in mainland Ecuador and has not been detected to date elsewhere in the Western Hemisphere (Fig. 5B). Our previous study of multiple locations in Ecuador found a homogeneous population with respect to TpiI4 with the same haplotype frequency profile in collections from the three ecological regions (Coast, Andes, Amazon) and different plant hosts (soft corn, hard corn, and rice)^42^. These observations indicate that the differences between populations on the two Galápagos islands are unlikely to be due to differences in habitats or host plants. Instead, it appears that fall armyworm movements between San Cristóbal and Santa Cruz islands are sufficiently restricted as to allow divergence in haplotype composition and frequency.

Consistent with this scenario is the pattern of genetic variation observed between Ecuador locations. Haplotype diversity and in particular nucleotide diversity is higher in fall armyworm from the mainland, intermediate in San Cristóbal, and lowest in Santa Cruz (Fig. 6). This is what would be expect if there is limited movement of a relatively small number of fall armyworm from the mainland to San Cristóbal, and very restricted subsequent exchanges of fall armyworm with neighboring Santa Cruz. Additional evidence for such behavior is that the TpiI4 haplotype profile of Ecuador appears to be more similar to that found in the rest of South America than fall armyworm in San Cristóbal to the nearby island of Santa Cruz. This observation supports earlier studies suggesting migration to and between islands may be problematic for fall armyworm. Substantial differences in haplotype profiles between neighboring islands of the Lesser Antilles were observed indicating that the Caribbean Sea acted as a barrier against the mixing of populations between the two Americas^38^. We now have evidence for similar restrictions in migration associated with the approximately 100 km of water separating the islands of San Cristóbal and Santa Cruz. These data suggest that even with favorable winds, fall armyworm has difficulty finding relatively small land masses in an ocean environment.

Long-term surveillance and haplotype analysis of the Galápagos fall armyworm could provide important insights into migratory moth behavior in ocean and island environments. If natural migration is as restricted as our data suggests, then populations between the islands should continue to diverge and more extensive differences in the haplotype profiles would be predicted. If trade is the primary means of fall armyworm movement from the mainland to the Galápagos, then the population in San Cristóbal should consistently be the most similar genetically to fall armyworm on the mainland. These hypotheses are easily testable and such studies would contribute to our understanding of how water barriers impact the movements of migratory moth pests.

## Methods

### Specimen collections and DNA preparation

Fall armyworm were collected from corn (hard) host plants on two islands in the province of the Galápagos during August 2018. Collections from San Cristóbal were made in the parish of El Progreso while those from Santa Cruz were from the parishes of Bellavista and Santa Rosa. Collections were limited to 1–2 larvae per plant. Fall armyworm from Argentina were from previously described collections^21^, but with additional specimens analyzed from storage. Other collections from Ecuador, the United States, and South America were previously described (Table 1). Specimens were placed in ethanol and stored at room temperature. DNA preparation was as describe previously^29^. A portion of each specimen was excised and homogenized in a 5-ml Dounce homogenizer (Thermo Fisher Scientific, Waltham, MA, USA) in 800 µl Genomic Lysis buffer (Zymo Research, Orange, CA, USA) and incubated at 55 °C for 5–30 min. Debris was removed by centrifugation at 10,000 rpm for 5 min. The supernatant was transferred to a Zymo-Spin III column (Zymo Research, Orange, CA, USA) and processed according to manufacturer’s instructions. The DNA preparation was increased to a final volume of 100 µl with distilled water. Genomic DNA preparations of fall armyworm samples from previous studies were stored at − 20 °C. Species identity was initially determined by morphology and confirmed by sequence analysis of the COIB region.

**Table 1:** Fall armyworm sampling site information for collections described in this study. Letters in parenthesis refer to map locations in Fig. 1.

Region	Province/State	Landmark	Host plant	References
Ecuador (Galapagos)	Galápagos	San Cristóbal	Corn	This paper
	Galápagos	Santa Cruz	Corn	This paper
Ecuador (Mainland)	Azuay (a)	Paute	Corn	^42^
	Bolivar (b)	San Miguel	Corn	^42^
	Imbabura (c)	Multiple	Corn	^42^
	Loja (d)	Multiple	Corn	^42^
	Manabi (e)	Portoviejo	Corn	^42^
	Morona-Santiago (f)	Multiple	Corn	^42^
	Napo (g)	Tena	Corn	^42^
	Sucumbios (h)	Multiple	Corn	^42^
	Guayas (i)	Multiple	Rice	^42^
	Loja (j)	Multiple	Rice	^42^
	Manabi (k)	Rocafuerte	Rice	^42^
South America	Argentina	Multiple	Multiple	^21^
	Brazil	Multiple	Multiple	^25^
	Peru	Lima	Corn	^38^
	Bolivia	Cotoca	Corn	^38^
United States	Texas	Multiple	Multiple	^14,28^
	Florida	Multiple	Multiple	^14,28^

### PCR amplification and DNA sequencing

PCR amplification for all segments was performed as previously described^29^. In short, a 30-µl reaction mix containing 3 µl 10 × manufacturer’s reaction buffer, 1 µl 10 mM dNTP, 0.5 µl 20-µM primer mix, 1 µl DNA template (between 0.05–0.5 µg), 0.5-unit Taq DNA polymerase (New England Biolabs, Beverly, MA). The thermocycling program was 94 °C (1 min), followed by 33 cycles of 92 °C (30 s), 56 °C (45 s), 72 °C (45 s), and a final segment of 72 °C for 3 min. Typically 96 PCR amplifications were performed at the same time using either 0.2-ml tube strips or 96 well microtiter plates. All primers were obtained from Integrated DNA Technologies (Coralville, IA) and are mapped in Fig. 1. Amplification of the COIB segment typically used the primer pair *924F* (5′-TTATTGCTGTACCAACAGGT-3′) and *1303R* (5′- CAGGATAGTCAGAATATCGACG-3′). When necessary nested PCR was used in which the first PCR was performed using primers *891F* (5′-TACACGAGCATATTTTACATC-3′) and *1472R* (5′-GCTGGTGGTAAATTTTGATATC-3′) followed by a second PCR using the internal primers *924F* and *1303R*. Amplification of the *Tpi* exon–intron segment used the primers *412F* (5′- CCGGACTGAAGGTTATCGCTTG -3′) and *1140R* (5′-GCGGAAGCATTCGCTGACAACC-3′) to produce a variable length fragment due to insertion and deletion mutations. Nested PCR was again used when needed with the first PCR done with primers *634F* (5′-TTGCCCATGCTCTTGAGTCC-3′) and *1166R* (5′-TGGATACGGACAGCGTTAGC-3′) and the second PCR using the internal primers *412F* and *1140R*.

DNA sequencing and analysis of the PCR products were performed as previously described^29^. Fragments were visualized using 1.8% agarose horizontal gel electrophoresis and the DNA stain GelRed (Biotium, Hayward, CA) in 0.5 × Tris–borate buffer (TBE, 45 mM Tris base, 45 mM boric acid, 1 mM EDTA pH 8.0) and isolated using Zymo-Spin I columns (Zymo Research, Orange, CA) according to manufacturer’s instructions. DNA sequencing was performed by Genewiz (South Plainfield, NJ). DNA alignments and consensus building were performed using MUSCLE (multiple sequence comparison by log-expectation), a public domain multiple alignment software incorporated into the Geneious Pro 10.1.2 program (Biomatters, New Zealand, http://www.geneious.com)^43^. Phylogenetic trees were graphically displayed in a neighbor-joining (NJ) tree analysis also included in the Geneious Pro 10.1.2 program^44^. Phylogenetic networks were estimated by the TCS statistical parsimony algorithm^45^ incorporated in the software program PopArt^46^.

### Characterization of the *CO1* and *Tpi* gene segments

The genetic markers identifying strains are all single nucleotide substitutions and their nomenclature and descriptions are as previously described ^29^. Sites in the *COI* gene are designated by an "m" (mitochondria) while *Tpi* sites are designated "g" (genomic). This is followed by the DNA name, number of base pairs from the predicted translational start site (*COI*), 5′ start of exon (*Tpi*), or 5′ start of the intron (TpI4) and the nucleotides observed using IUPAC convention (R: A or G, Y: C or T, W: A or T, K: G or T, S: C or G, D: A or G or T). The gTpi183Y site is on the fourth exon of the predicted *Tpi* coding region (Fig. 1B). The C-strain allele (*Tpi*C) is indicated by a C_183_ and the R-strain (*Tpi*R) by T_183_^24^. The *Tpi* gene is located on the *Z* sex chromosome that is present in one copy in females and two copies in males. Heterozygosity for two *Tpi* alleles will result in the DNA sequence chromatograph displaying overlapping curves at polymorphic sites. Heterozygosity at the strain defining gTpi183Y site was always limited to Y (C from C-strain and T from R-strain) and was denoted as *Tpi*H or hybrid^29^.

The *Tpi* intron segment (TpiI4) immediately downstream of TpiE4 includes the first ¾ of a variable length intron with multiple insertions and deletions (indels). TpiI4 is usually approximately 170 bp in length, which can increase to approximately 400 bp. The segment was initially sequenced with primer 891F, with 1140R used for 2nd strand sequence confirmation in cases of ambiguity. This segment was chosen for analysis because it empirically had the most consistent sequence quality with the given primers. Sequences showing intron polymorphism due to heterozygosity were not further analyzed.

### Calculation of haplotype numbers

Analysis of haplotypes was as previously described^29^, and repeated as follows. The mitochondrial *COI* markers are calculated directly as the number of specimens exhibiting the *COI* haplotypes divided by total specimens. Because *Tpi* is a sex-linked nuclear gene on the *Z*-chromosome, it is present in two copies in males (ZZ) and one copy in females (ZW). Therefore, specimens with a TpiC or TpiR haplotype could be either a homozygous male or hemizygous female. We assumed a 1:1 sex ratio^47^ for the larval specimens so that the average number of *Tpi* genes per specimen is given as 1.5 as calculated: (2 in males + 1 in females)/2. Based on this reasoning the number of TpiC and TpiR specimens were multiplied by 1.5 to estimate the number of chromosomes carrying each marker. In comparison, TpiH specimens are heterozygous and so carried one of each marker. The estimated number of TpiC chromosomes was calculated by 1.5TpiC + TpiH, and TpiR chromosomes = 1.5TpiR + TpiH.

### Data and statistical analyses

The *COI*-h and TpiI4 haplotype profiles for different populations were compared by *chi*-square analysis for homogeneity, with the null hypothesis being that the compared populations are identical. Quantification of genetic variability was made by calculations of haplotype diversity (Hd) and nucleotide diversity (Pi), which is a measure of the average number of nucleotide differences between randomly chosen sequences from a population. These were performed using the DNAsp software package^48^. Haplotype profiles were compared by correlational analysis using Pearson correlation r. Statistical analyses were conducted using GraphPad Prism version 7.00 for Mac (GraphPad Software, La Jolla, CA USA). Generation of graphs were done using Excel and PowerPoint (Microsoft, Redmond, WA, USA). Geographical maps were generated using QGIS version 2.18.2 (Open Source Geospatial Foundation).

### HYSPLIT air trajectory projections

Air transport trajectories for Ecuador were estimated using the Hybrid Single Particle Lagrangian Integrated Trajectory Model at the Air Resources Laboratory (ARL) READY web site run by NOAA (http://ready.arl.noaa.gov/HYSPLIT.php^49^. Source locations (latitude, longitude) were in Loja province (− 4.02, − 79.27), Manabi province (− 1.10, − 80.43), San Cristóbal Island (− 0.87, − 89.43), and Santa Cruz Island (− 0.59, − 90.32). Projections were made for the 30-day period beginning June 1, 2018, at 800 m above ground level (AGL). The 30-day period was chosen to provide a representative sampling of wind projections during June. Flights were projected beginning 18:00 h and extending for 12, 72, or 96 h continuous flight. Flight projections were repeated every 24 h for the 30-day period, resulting in 30 trajectories for each source. The trajectories were averaged and displayed as a frequency distribution with percentages reflecting the likelihood of a projection entering a given grid.

### Wind climatology maps

The generation of wind vector maps was as previously described^38^ and repeated here. Monthly average wind velocity (vector mean wind speed and wind direction) data were from the 1981–2010 base period derived from NCEP-NCAR reanalysis of monthly zonal (i.e., westerly) and meridional (i.e., southerly) wind velocity components at a pressure-height of 850 mb (hPa) (source: National Oceanic and Atmospheric Administration, NOAA, and the National Centers for Environmental Prediction, NCEP). The analyzed maps were obtained online from the International Research Institute for Climate and Society (Earth Institute, Columbia University, New York, NY; http://iridl.ldeo.columbia.edu/maproom/Global/Climatologies/Vector_Winds.html).

## Data Availability

All data generated or analyzed during this study are included in or referenced in this article. The relevant DNA sequences from Ecuador are deposited into GenBank or identical to previously deposited sequences (MT628749-MT628900) and include the TpiI4 haplotypes e01 (MT628803), e02 (MT628802), e08 (MT628809), e10 (MT628801), e13 (MT628812), e16 (MT628808).
